# Vaseline in Nasal Discharges

**Published:** 1877-05-20

**Authors:** 


VASELINE IN NASAL DISCHARGES.—Dr. H. A. Dubois, of California, says in *Medical Record*, that in discharges resulting from ulcerations of the septum of the nose, the treatment found most effectual has been the use, night and morning, of vaseline, with five grains of salacylic acid added to the ounce, introduced into the affected nostril by a camel's hair pencil, or upon a little cotton wound around the end of a stick, using internally minute doses of corrosive sublimate, with some preparation of iron.

